# Frequency of Depression and Anxiety Symptoms in Surgical Hospitalized Patients

**DOI:** 10.7759/cureus.4141

**Published:** 2019-02-26

**Authors:** Shahid H Mirani, Dharmoon Areja, Syeda Suman Gilani, Amber Tahir, Murk Pathan, Suman Bhatti

**Affiliations:** 1 Surgery, Ghulam Mohammad Mahar Medical College and Hospital, Sukkur, PAK; 2 Surgery, Ghulam Muhammad Mahar Medical College and Hospital, Sukkur, PAK; 3 Internal Medicine, Dow University of Health Sciences, Karachi, PAK; 4 Internal Medicine, Ghulam Mohammad Mahar Medical College and Hospital, Sukkur, PAK; 5 Internal Medicine, Ghulam Muhammad Mahar Medical College and Hospital, Sukkur, PAK

**Keywords:** hospital anxiety and depression scale, surgical patients, anxiety, depression

## Abstract

Background

Patients hospitalized for surgeries and those with chronic remitting health conditions develop hospital-induced anxiety and depression. Unfamiliar hospital environment, insufficient privacy, exposure to strange instruments, financial concerns, disease stress, and prolonged hospital stay contribute to this anxiety. The aim of this study was to assess the frequency of depression and anxiety in surgical patients.

Material and methods

This observational study was conducted among 50 patients hospitalized for 10 days or more in the surgical unit. All patients completed the Hospital Anxiety and Depression Scale (HADS). It has seven items for anxiety and depression each. Each item scores 0-3. A subscale score >8 denotes anxiety or depression. Data were entered and analyzed using SPSS v.20. Mean and standard deviation (SD) were calculated for descriptive data, and frequencies and percentages were calculated for categorical data.

Results

On the HADS, the mean ± SD score of anxiety was 11.84 ± 4.16 and that of depression was 12.78 ± 4.16. There were 64% severely anxious and 74% severely depressed hospitalized patients. More patients with less than two weeks of hospital stay were severely depressed than severely anxious (72.7% vs. 36.3%). There were 20% patients with moderate anxiety and depression with hospital stay longer than three weeks and 80% with severe anxiety and depression.

Conclusion

There is a high incidence of anxiety and depression in surgical patients. Patients at risk of developing these symptoms must be identified and psychological care should be provided to them.

## Introduction

In patients hospitalized for surgeries and those with chronic remitting health conditions, development of anxiety and depression has been studied [1-2]. Psychological disorders are mostly due to inadequate adjustment to the unfamiliar hospital environment, insufficient privacy, exposure to strange instruments, financial concerns, and disease stress [3]. It is also associated with a higher incidence of unsatisfactory treatment outcomes [4]. In some instances, anxiety and depression have been morbid enough to result in increased morbidity, readmission, and psychiatric diagnosis after discharge [1,5-6].

Even then, there are not any vigorous instruments and strategies to identify patients who are at the risk of developing hospital-induced anxiety. Patient anxiety has a direct association with patient behavior. Anxious patients can be more difficult for the nursing staff or can be apprehensive enough to not cooperate with history taking or following instructions. Anxious surgical patients require higher doses of anesthesia as well as higher dose and longer duration of post-surgical analgesia [7].

The Hospital Anxiety and Depression Scale (HADS) has been utilized considerably over time to screen hospitalized patients for the development of signs of anxiety and depression. It has been repeatedly validated applicability in the general population [8-10]. In a large longitudinal population-based study from the United Kingdom, “moderate-to-severe” anxiety was seen in 19% women and 12.5% men. Women and men with any level of depression were 17.2% and 15.4%, respectively. “Moderate-to-severe” depression was seen in 6.9 % women and men [11]. In a Pakistani study with tuberculosis patients, 46.3% were depressed and 47.2% were anxious on the HADS scale [12]. Preoperative anxiety has been reported in 62% of surgical patients [13].

Based on this scenario, this study utilized the HADS to screen surgical patients to assess the frequency of depression and anxiety.

## Materials and methods

It was an observational study in which 50 participants were recruited who were hospitalized in the surgical department of Ghulam Muhammad Mahar Hospital, Sukkur for 10 days or more. After attaining informed consent, gender, age, and duration of hospital stay in days were noted for all patients. HADS was administered in all patients. All patients included in the study were post-operative. HADS is a 14-item inventory - seven to assess depression and seven to assess anxiety. All questions were to be responded on a four-point Likert scale. After summing up scores, a total score of 0-7 was taken as “no depression/anxiety”, 8-10 was taken as “moderate depression/anxiety”, and 11-21 was taken as “severe depression/anxiety”. The mean reliability and internal consistency of HADS-anxiety was 0.83 and that of HADS-depression is 0.82 [10].

All data were entered and analyzed using SPSS version 22. Mean and standard deviation (SD) were calculated for descriptive variables including age, length of hospital stay, and anxiety and depression scores. Frequencies and percentages were calculated for stratified variables.

## Results

Out of the 50 patients who completed the study, there were 15 men (30%) and 35 women (70%). Their mean age was 39.60 ± 14.35 years (range: 23-58 years), and their mean duration of stay in the hospital was 16.66 ± 3.00 days (range: 11-21 days). The mean anxiety score of the patients was 11.84 ± 4.16, and the mean depression score was 12.78 ± 4.16 (range: 0-21).

When the anxiety and depression score was categorized as no anxiety/depression, moderate, and severe, it was seen that 64% of hospitalized patients were “severely anxious” and 74% of hospitalized patients were “severely depressed”, as shown in Table 1.

**Table 1:** Hospital anxiety and depression scale score for anxiety and depression (N = 50)

	FREQUENCY	PERCENTAGE
Anxiety		
No anxiety	7	14%
Moderate anxiety	11	22%
Severe anxiety	32	64%
Depression		
No depression	5	10%
Moderate depression	8	16%
Severe depression	37	74%

There were more non-depressed women than men (11.4% vs. 6.7%); however, there were also more severely depressed women than men (80% vs. 60%). Severe anxiety was seen in 53.3% men and 68.5% women. In the younger age group of patients, 66.7% were severely depressed and 63.3% were severely anxious. All of the patients of more than 60 years of age were severely depressed; 75% of these had severe anxiety as well. More patients with less than two weeks of hospital stay were severely depressed than severely anxious (72.7% vs. 36.3%). Approximately 20% of the patients presented with moderate anxiety and depression with hospital stay longer than three weeks and 80% with severe anxiety and depression. The stratification of anxiety and depression in terms of gender, age, and duration of hospital stay is shown in Table 2.

**Table 2:** Stratification of the HADS score of depression and anxiety in terms of patient gender, age, and length of hospital stay (N = 50) HADS: hospital anxiety and depression scale

	GENDER		AGE IN YEARS			DURATION OF HOSPITAL STAY IN WEEKS		
	Male	Female	< 40	40-60	> 60	< 2	2-3	> 3
Depression								
Normal	1 (6.7%)	4 (11.4%)	5 (16.7%)	0	0	2 (18.1%)	3 (8.8%)	0
Borderline	5 (33.3%)	3 (8.5%)	5 (16.7%)	3 (18.7%)	0	1 (9.1%)	6 (17.6%)	1 (20%)
Abnormal	9 (60%)	28 (80%)	20 (66.7%)	13 (81.2%)	4 (100%)	8 (72.7%)	25 (73.5%)	4 (80%)
Anxiety								
Normal	3 (20%)	4 (11.4%)	4 (13.3%)	3 (18.7%)	0	3 (27.3%)	4 (11.7%)	0
Borderline	4 (26.7%)	7 (20%)	7 (23.3%)	3 (18.7%)	1 (25%)	4 (36.3%)	6 (17.6%)	1 (20%)
Abnormal	8 (53.3%)	24 (68.5%)	19 (63.3%)	10 (62.5%)	3 (75%)	4 (36.3%)	24 (70.5%)	4 (80%)

**Figure 1:**
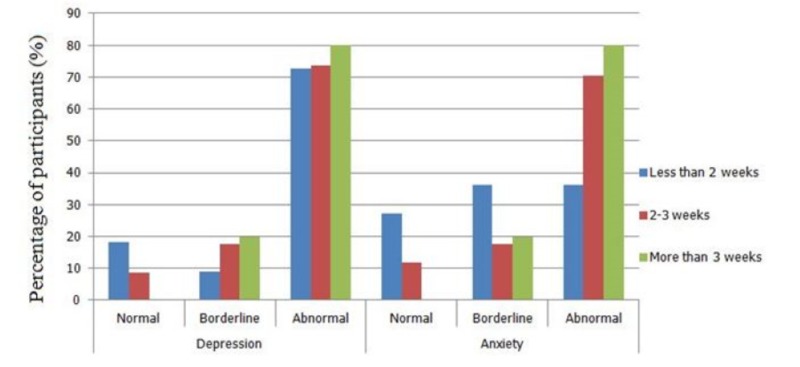
Severity of anxiety and depression according to the HADS score based on the duration of hospital stay HADS: hospital anxiety and depression scale

The severity of anxiety and depression according to the HADS scoring criteria stratified according to the duration of hospital stay is shown in Figure 1.

## Discussion

On the HADS, there were 64% severely anxious and 74% severely depressed hospitalized patients. More patients with less than two weeks of hospital stay were severely depressed than severely anxious. The frequency of anxiety and depression in patients admitted for more than three weeks was similar.

In the past few years, there has been an increasing interest in the psychosocial health of chronically ill and hospitalized patients. The paucity in the existing literature is still a concern, particularly from the South Asian countries. In a local study, moderate to severe depression was seen in people suffering from anemia, diabetes, hypertension, and asthma [14]. Anxiety and depression have been reported from 20% to 60% in the general population of Pakistan [15]. The percentages in this study with hospitalized patients are higher. Among the non-hospitalized patients with chronic pains, 53% of them were reported to be depressed and 44% were anxious [16].

One major limitation of the study is that it lacks a control group for inference of relationship. However, its strength is that the instrument used in this study is a strong one; it has been previously validated. Although the overall sample is small, this study managed to recruit almost all of the eligible patients; hence, it is representable and generalizable sample size.

On HADS, preoperative anxiety in 61% of adult surgical patients was seen with fear of complications, female gender, and lack of information on the surgical procedure being the strong factors [17]. When anxiety and depression were assessed in women undergoing hysterectomy, it was seen that 62% were anxious, 29% were borderline anxious, 36% were depressed, and 46% were borderline depressed. Although hysterectomy itself is a stress-inducing procedure for women, none of the women under study were receiving any psychological aid [18].

Psychiatric symptoms of anxiety and depression in hospitalized surgical patients as well as hospitalized and non-hospitalized patients with major chronic illnesses are prevalent yet undermined. This area of research is still underdeveloped and requires attention. Large-scale, case-control studies are needed to strengthen the relationship of long hospital admissions, complicated and chronic illnesses with the development of psychiatric illnesses. Furthermore, studies should be conducted to highlight any impact of this anxiety and depression in patient outcome especially in cases of surgical patients. Patients prone to the development of anxiety and depression must be advised psychological care.

## Conclusions

Surgical patients have high incidence of anxiety and depression. Longer duration of hospital stay plays a crucial role in this manner. Large-scale, case-control studies are needed to strengthen the relationship of long hospital admissions, complicated and chronic illnesses with the development of psychiatric illnesses and the impact of these psychiatric illnesses on disease outcome. These patients should be offered psychological care.

## References

[1] Daratha KB, Barbosa-Leiker C, H Burley Mm (2012). Co-occurring mood disorders among hospitalized patients and risk for subsequent medical hospitalization. Gen Hosp Psychiatry.

[2] Elliott PC, Murphy BM, Oster KA, Le Grande MR, Higgins RO, Worcester MU (2010). Changes in mood states after coronary artery bypass graft surgery. Eur J Cardiovasc Nurs.

[3] Chhari N, Mehta SC (2016). Stress among patients during hospitalization: a study from Central India. Ntl J Community Med.

[4] Ghoneim MM, O’Hara MW (2016). Depression and postoperative complications: an overview. BMC Surg.

[5] Gasse C, Laursen TM, Baune BT (2014). Major depression and first-time hospitalization with ischemic heart disease, cardiac procedures and mortality in the general population: a retrospective Danish population-based cohort study. Eur J Prev Cardiol.

[6] Gerson S, Mistry R, Bastani R (2004). Symptoms of depression and anxiety (MHI) following acute medical/surgical hospitalization and post-discharge psychiatric diagnoses (DSM) in 839 geriatric US veterans. Int J Geriatr Psychiatry.

[7] Pritchard MJ (2011). Using the hospital anxiety and depression scale in surgical patients. Nurs Stand.

[8] Bjelland I, Dahl AA, Haug TT, Neckelmann D (2002). The validity of the hospital anxiety and depression scale. An updated literature review. J Psychosom Res.

[9] Zigmond AS, Snaith RP (1983). The hospital anxiety and depression scale. Acta Psychiatr Scand.

[10] Bjelland I, Dahl AA, Haug TT, Neckelmann D (2002). The validity of the hospital anxiety and depression scale: an updated literature review. J Psychosom Res.

[11] Breeman S, Cotton S, Fielding S, Jones GT (2015). Normative data for the hospital anxiety and depression scale. Qual Life Res.

[12] Husain MO, Dearman SP, Chaudhry IB, Rizvi N, Waheed W (2008). The relationship between anxiety, depression and illness perception in tuberculosis patients in Pakistan. Clin Pract Epidemiol Ment Health.

[13] Jafar MF, Khan FA (2009). Frequency of preoperative anxiety in Pakistani surgical patients. J Pak Med Assoc.

[14] Godil A, Mallick MS, Adam AM (2017). Prevalence and severity of depression in a pakistani population with at least one major chronic disease. J Clin Diagn Res.

[15] Ahmed B, Enam SF, Iqbal Z, Murtaza G, Bashir S (2016). Depression and anxiety a snapshot of the situation in Pakistan. Int J Neurosci Behav Sci.

[16] Ahmed S, Haque SN, Hamirani M, Haque SM, Sohail S, Munir F (2013). Frequency of anxiety and depression in patients with pain visiting pain clinic. Pak J Surg.

[17] Mulugeta H, Ayana M, Sintayehu M, Dessie G, Zewdu T (2018). Preoperative anxiety and associated factors among adult surgical patients in Debre Markos and Felege Hiwot referral hospitals, Northwest Ethiopia. BMC Anesthesiol.

[18] Raza N, Waqas A, Jamal M (2015). Post-operative anxiety, depression and psychiatric support in patients undergoing hysterectomy: a cross-sectional survey. J Pak Med Assoc.

